# Effect of Melamine Formaldehyde Resin Encapsulated UV Acrylic Resin Primer Microcapsules on the Properties of UV Primer Coating

**DOI:** 10.3390/polym16162308

**Published:** 2024-08-15

**Authors:** Yuming Zou, Yongxin Xia, Xiaoxing Yan

**Affiliations:** 1 Co-Innovation Center of Efficient Processing and Utilization of Forest Resources, Nanjing Forestry University, Nanjing 210037, China; zou_yuming@njfu.edu.cn (Y.Z.); xiayongxin@njfu.edu.cn (Y.X.); 2 College of Furnishings and Industrial Design, Nanjing Forestry University, Nanjing 210037, China

**Keywords:** microcapsules, UV coatings, self-healing

## Abstract

Ultra-Violet (UV) coatings are widely adaptable of substrates and produce low emissions of volatile organic compounds. UV coatings can extend service life by adding self-healing microcapsules that restore integrity after sustaining damage. In this study, UV coating was used as a core material; microcapsules were produced and added to the UV coating to enhance its self-healing property, providing a good protection for both the UV coating and the substrate. UV primer microcapsules were prepared with UV primer as the core material and melamine formaldehyde resin as the wall material. The UV primer containing more than 98.0% solids content was mainly composed of epoxy acrylic resin, polyester acrylic resin, trihydroxy methacrylate, trimethyl methacrylate, and photo initiator. The preparation process of the UV primer microcapsules was optimized. Further, the UV coating was prepared with better UV primer microcapsules, and the effects of the UV primer microcapsules alongside the comprehensive properties of the coating were studied. The best preparation process for the UV primer microcapsules was as follows: the wall-core mass ratio was 1:0.50, Triton X-100 and Span-20 as emulsifiers with an HLB value of 10.04, the microcapsule reaction temperature was 70 °C, and the reaction time of the was 3.0 h. When the quantity of the UV primer microcapsules increased in the coating, color difference Δ*E* of the coating increased, gloss decreased, transmittance decreased, elongation at break increased and then decreased, roughness increased, and self-healing rate first increased and then decreased. When the addition of the UV primer microcapsules reached 2.0%, the color difference Δ*E* of the coating was 1.71, the gloss was 106.63 GU, the transmittance was 78.80%, the elongation at break was 3.62%, the roughness was 0.204 μm, and the self-healing rate was 28.56%, which were the best comprehensive properties of the UV primer. To improve the comprehensive properties of the UV coatings, the UV coatings were modified by a microcapsule technology, which gave the UV coatings a better self-healing property. The application range of microcapsules for the UV coatings was broadened. Based on the previous research of microcapsules in UV coatings, the results further refined the study of the effects of adding self-healing microcapsules to UV coatings using the UV coating itself as the core material.

## 1. Introduction

As a natural material, wood is widely used in the furniture industry [1,2,3,4,5]. Various modification technologies have been applied to furniture surfaces to provide the surfaces with superior properties [6,7,8,9,10]. Applying coatings is considered as a direct, cheap, and high-quality surface technology that can protect the surfaces of wooden furniture from the external environment to a certain extent [11,12,13]. However, various properties, such as a coating adhesion and service life, limit the effectiveness of the coatings on the surface of wooden furniture. These properties are influenced by factors such as humidity and temperature, and several types of defect, such as cracking, blistering, and complex maintenance problems, arise in relation to the coating [14,15]. Further improvement of the self-healing property of coating on the surface of wooden furniture is needed to increase the utilization of wood, increase the service life of wood, and thus reduce the waste of wood [16,17,18]. Microcapsule technology, a commonly used coating modification method, is both low cost and has a diverse range of functions. By modifying coatings with microcapsules, the wooden surface can benefit from these functions [19,20]. Microcapsules are a class of tiny particles with a wall-core structure, which function the wall material by encapsulating a core material [21,22,23]. In this study, UV coatings were modified with microcapsule technology to enhance the comprehensive properties of the UV coatings, to give the UV coatings a better self-healing function, and to broaden the application range of UV coating microcapsules [24,25]. UV coating microcapsules with the self-healing property were added to the UV coatings to create self-healing coatings, lay a test foundation, improve quality of life, extend the coatings and substrate service life, and promote the green and healthy development of a coating industry, which is important for sustainable development. UV coatings have the required properties to act as the core material to form the microcapsules [26,27]. When the UV primer was damaged, the core material that flowed out after the microcapsules ruptured was consistent with the coating material, showing that the core material can be well fused with the coating material. After a period of irradiation time due to natural light striking the coating, microcracks in the coating can be healed effectively. For furniture with complex surfaces, the UV coatings are normally applied by spraying and other multi-angle application methods to ensure that the furniture is uniformly coated. The curing time is also different; in this paper, we used a single fixed direction UV lamp as the irradiation source for the coating process. Usually, a multi-angle UV lamp is used as the irradiation source, and in more complex situations, manually adjusting the single UV lamp is required. Requirements for the properties of self-healing microcapsules will also be discussed.

In the 1970s, microcapsule technology matured and its use gradually expanded into biomedical [28], food [29], cosmetics [30], coatings [31], and other fields. Recently, with the continuous development of microcapsule technology, the exploration of new functions of microcapsule technology has become a hot research topic. Microcapsules have a wide range of applications in the coating industry. The application of microcapsules gives coatings distinctive properties, such as fire resistance, antibacterial effects, and self-healing abilities. UV coatings have various excellent properties, but lack the ability to self-heal [32,33,34]. Li et al. [35] developed UV-triggered self-healing SiO_2_/polydopamine (PDA) hybrid microcapsules with enhanced UV shielding and compatibility with epoxy coatings. The microcapsules contain a liquid core with a blend of epoxy resins (E-51 and A1815) and a UV initiator (PI6992) as well as a novel UV-shielded SiO_2_/PDA hybrid shell with an ultrathin PDA layer uniformly embedded in a mesoporous structure of a shell. As a UV absorber, PDA imparts a UV-shielding hybrid wall to the SiO_2_/PDA hybrid microcapsules and protects the core material from undesirable UV curing behaviors prior to self-healing in spaces with high amounts of UV radiation. The epoxy resin coating containing SiO_2_/PDA microcapsules retained a high degree of self-healing ability after 192 h of aging under UV irradiation, which was attributed to its enhanced UV shielding ability and compatibility. At present, most of these studies focus on the enhancement of curing rates of UV coatings, the improvement of mechanical properties, and the enhancement of multifunction properties, etc., but research on how to carry out self-healing of UV coatings after the microcracks in practical applications has not received extensive attention. Traditionally, self-healing microcapsules use exogenous substances as the core material, but whether the exogenous substances have good compatibility with a surrounding environment after the microcapsules rupture and outflow, and whether the chemical reaction reduces the self-healing rate, have yet to be discussed. Therefore, the use of materials consistent with the coating being self-healing and what material to be used as the core material for the preparation of the self-healing microcapsules has a certain research value. Considering that microcracks can occur across the coating, microcapsules can be effective as a self-healing agent. The choice of the core material for the microcapsules is particularly important. This paper will test the possibility of using the coating itself as the core material for self-healing microcapsules and will investigate the effects of such microcapsules on various properties of the coating. In the future, research on using the coating itself as the core material for self-healing microcapsules can be expanded to other coatings with self-healing properties. The UV primer with the self-healing property can be applied to woods, metals, and other substrates that are easily deformed by external factors. The UV primer with an addition of UV primer microcapsules can be directly or indirectly applied to various types of substrate surfaces.

Melamine formaldehyde resin was used as the wall material. The UV primer was used as the core material. Orthogonal tests were designed to investigate the effects of a wall-core mass ratio, emulsifier HLB value, microcapsule reaction temperature and a microcapsule reaction time on yield, overall encapsulation rate, microscopic morphology, and the chemical composition of the UV primer microcapsules. The results show that the most influential factor was the wall-core mass ratio. By setting a suitable wall-core mass ratio for single-factor tests, a better process for preparing the UV primer microcapsules was obtained. Then, the UV primer and better UV primer microcapsules were mixed to make the coating, and a study was carried out to characterize the effects that the UV primer microcapsules had on the comprehensive properties of the UV primer.

## 2. Materials and Methods

### 2.1. Materials and Equipment

Molds for preparing coating were 50 mm × 20 mm × 15 mm. Test materials are shown in Table 1. The coating and the core material of microcapsules used in the test was UV primer, which was comprised of epoxy acrylic resin, polyester acrylic resin, trihydroxy methacrylate, trimethyl methacrylate, photo initiator, defoamer, levelling agent, etc. The UV primer had more than 98.0% solid content.

The test equipment is shown in Table 2. A single-lamp curing machine with a UV mercury lamp with a light intensity of 80–120 W/cm^2^ was used. The wavelength range of the UV irradiation released was 250–420 nm.

### 2.2. Process of UV Primer Microcapsules Preparing

Four factors, the wall-core mass ratio, the emulsifier HLB value, the microcapsule reaction temperature, and the microcapsule reaction time during the UV primer microcapsules preparation process, were selected for the orthogonal test to determine the most significant factor among them, and that factor was then used as the variance for the single-factor test. The orthogonal test design is shown in Table 3. The orthogonal test detail list, the orthogonal test material list, and the single-factor test material list are shown in Table 4, Table 5, and Table 6, respectively. The emulsifier HLB value was calculated in Equation (1). *H* was the HLB value. *P*_1_ was the Triton X-100 proportion. *P*_2_ was the Span 20 proportion.

*H* = *P*_1_ × 13.4 + *P*_2_ × 8.6 (1)

The process of preparing the UV primer microcapsules was derived from our previous research. What follows is the preparation process for sample 2 to illustrate the process [36]: 

Process of wall material preparation: the molar ratio of formaldehyde and melamine formaldehyde resin was 3.5:1.0; 4.00 g of melamine formaldehyde resin and 9.01 g of formaldehyde resin were weighed in a beaker. The water bath temperature was 60 °C and the rotational speed was 700 rpm [36]. An appropriate amount of melamine formaldehyde resin was dripped into the beaker at a set a pH of 9.0, and then a reaction was performed for 20 min to obtain a solution of the wall material. Then, the wall material was kept in a heat preservation standby until needed.

Process of core material preparation: 0.08 g of Triton X-100 and 0.22 g of Span-20 were weighed and combined with 78.90 mL ethanol. An emulsifier solution was obtained by stirring. Then, 4.40 g of the UV primer was added to the solution. The rotational speed was set to 700 rpm and the temperature was 60 °C. After the 70 min reaction, the core emulsion was obtained.

Process of the UV primer microcapsules preparation: the core emulsion was added to the wall materials in a holding state, and the water bath was adjusted to 70 °C. Citric acid monohydrate was added, reducing the pH to 4.0. The reaction was carried out for 2.0 h. After cooling and standing for 5 d, the product was rinsed and pumped several times through a circulating water vacuum pump using ethanol and deionized water, and the resulting solids were placed in an oven at 60 °C. The UV primer microcapsules were the powder obtained by drying.

### 2.3. Method of UV Primer Coating Preparing

The best UV primer microcapsules obtained from the single-factor test were added to the UV primer at rates of 0%, 2.0%, 4.0%, 6.0%, 8.0%, and 10.0%; the combined mass of the UV primer microcapsules and primer was 1.50 g. After mixing evenly, the UV primer was poured into coating preparation molds, and the mixed UV primer was naturally leveled off. Then the molds were put on the conveyor belt of the single-lamp curing machine, and the conveyor speed was adjusted to 0.05 m/s. The curing time was about 20 s. The cured coating was demolded and tested for optical properties, mechanical properties and the self-healing property. The UV primer without microcapsules was used as the blank control group. The above mixed primer with different ratios was coated onto glass plates by hand brushing to test the roughness of the coating surface. The UV primer microcapsules were mixed with the UV primer directly. The particle size of the UV primer microcapsules was very small and there was no chemical reaction, meaning that the UV primer microcapsules would not rupture.

### 2.4. Test and Characterization

#### 2.4.1. UV Primer Curing Test

When the UV primer microcapsules rupture, it is critical that the outflowed core material can achieve natural curing. The UV primer was applied to the glass plates. Curing the UV primer under natural light conditions on days of 1, 4, 7, and 14 after the application was recorded. The test could verify whether it is feasible to use the UV primer directly as the core material and whether it can be cured with the natural light after the core material flows out.

#### 2.4.2. Yield Rate and Encapsulation Rate

The yield rate was recorded as *P*, the mass of the prepared microcapsules after drying was recorded as *m*_1_, and the total mass of the components of the wall materials and core materials used in the preparation of the coating was recorded as *m*_2_. The yield was calculated as in Equation (2).

*P* = (*m*_1_/*m*_2_) × 100% (2)

The encapsulation rate was recorded as *P_e_* and the weight of the microcapsules was recorded as *m*_3_. After thorough grinding, the microcapsules were added to the ethanol and soaked for 24 h, and then placed in the water bath at 70 °C for 3.0 h. After completion of the reaction, the microcapsules were rinsed and pumped, and after filtration, the microcapsules were placed in an oven at 60 °C for drying. The mass of the dried remains was recorded as *m*_4_. The encapsulation rate was calculated using Equation (3).

*P_e_* = [(*m*_3_ − *m*_4_)/*m*_3_] × 100% (3)

#### 2.4.3. Microscopic Characterization

Optical microscope: The sample was placed on an optical microscope for observation. The microscope was set to the magnification level required for the test.

Scanning Electron Microscope (SEM): The sample was placed on a sample plate with an adhesive. After gold spraying, the sample was placed on an observatory inside the SEM for vacuuming and then observed once the air pressure complied with the experimental conditions [37].

#### 2.4.4. Chemical Composition

An infrared spectrometer was used to analyze the chemical composition of the prepared microcapsules and coating.

#### 2.4.5. Optical Properties

Color difference: A test method of GB/T 11186.3-1989 [38] was referred to, and a color difference meter was used to obtain the *L*, *a*, and *b* of the coating. *L* value represented the brightness value of the sample, *a* value represented the red-green value of the sample, and *b* value represented the yellow-blue value of the sample. The test data for the control coating were *L*_1_, *a*_1_, and *b*_1_, and the test data for the coating with the addition of microcapsules were *L*_2_, *a*_2_, and *b*_2_. Color difference Δ*E* was calculated according to Equation (4), where Δ*L* = *L*_2_ − *L*_1_, Δ*a* = *a*_2_ − *a*_1_, Δ*b* = *b*_2_ − *b*_1_, and the data of each group was measured three times and the average value was taken [39,40,41].

Δ*E* = [(Δ*L*)^2^ + (Δ*a*)^2^ + (Δ*b*)^2^]^1/2^ (4)

Gloss: The test method of GB/T 4893.6-2013 [42] was referred to, and a glossmeter was used to measure the gloss. The gloss was tested and recorded at three incidence angles (20°, 60°, and 85°) to compare differences [43].

Transmittance: The transmittance in a visible wavelength range was measured by a UV spectrophotometer. The transmittance referred to a beam of light through a medium, the actual arrival of light intensity, and the original light intensity of the ratio between, often expressed as a percentage [44].

#### 2.4.6. Mechanical Properties

Elongation at break: The coating was produced to a standard specification, and the ends of the coated sample were clamped using the clamping arms of a universal mechanical testing machine. The coating was stretched at a speed of 0.5 mm/min until it was broken. The elongation at break of the coating was calculated as shown in Equation (5), where *e* represented the elongation at break of the coating at a breaking point, *L*_0_ was the initial distance between the two clamping arms when the coating was stretched, and *L* was the distance between the two clamping arms when the coating was broken [45,46,47,48].

*e* = [(*L* − *L*_0_)/*L*_0_] × 100% (5)

Roughness: A roughness meter was used to test and record values to derive trends in the roughness between coatings [49].

#### 2.4.7. Self-Healing Property

A scalpel was used to cut a crack of about 15 mm in the coating, and the width of the widest part of the crack was measured using the measurement function of the supporting software of the optical microscope; the data were recorded as *W*_1_. After one week, the width was measured for a second time in the same position of the crack of the coating; the data were recorded as *W*_2_. The calculation method of the self-healing rate of the coating (*W*) is shown in Equation (6), which was used to compare the self-healing property between different coatings by self-healing rate.

*W* = [(*W*_1_ − *W*_2_)/*W*_1_] × 100% (6)

## 3. Results and Discussion

### 3.1. UV Primer Curing Results

**Figure 1:**
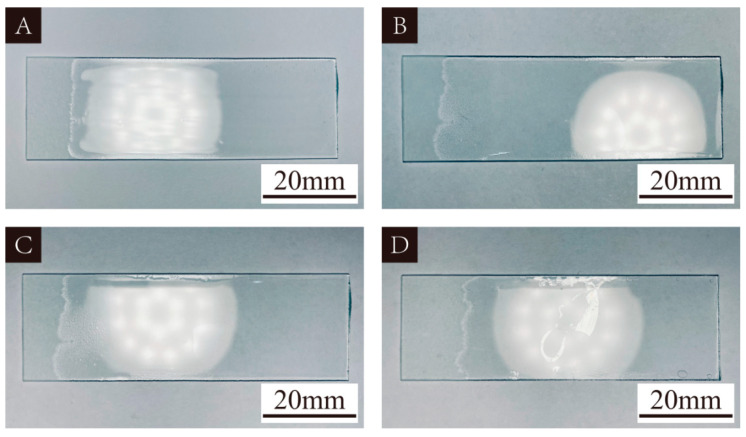
Curing of UV primer: (**A**) day 1, (**B**) day 4, (**C**) day 7, (**D**) day 14.

Figure 1 is the curing of the UV primer applied to the glass plate on days 1, 4, 7, and 14 under natural light irradiation. The UV primer used was transparent and had good fluidity. A trend of gradual curing of the UV primer could be seen, which proved that the UV primer could complete curing under natural light irradiation.

### 3.2. UV Primer Microcapsules

#### 3.2.1. Analysis of Yield Rate and Encapsulation Rate

Table 7 shows an analysis table of the yield rate results of the UV primer microcapsules produced by the orthogonal test. Among nine sets of microcapsules’ samples in the orthogonal test, sample 3 had the highest yield rate (*P*) of 32.22%, followed by sample 4 with the yield rate of 27.65% and sample 2 with a yield of 26.42%. The extreme difference in the results indicated that the wall-core mass ratio had the greatest effect on the yield of the UV primer microcapsules. For the yield of UV primer microcapsules, the levels of the four factors were A > D > B > C, and the recommended preparation process was A1B2C2D3. 

Table 8 shows the analysis table of the variance results of the yield of the UV primer microcapsules produced by the orthogonal test. The variance results were in line with the range results, and none of the four factors was significant.

Table 9 shows the analysis table of the results of the encapsulation rate (*P*_e_) of the UV primer microcapsules prepared by the orthogonal test. According to the range results, the four factors have the following levels of significance, A > B > D > C. Factor A, i.e., the wall-core mass ratio, was the factor with the largest effect on the encapsulation rate of the UV primer microcapsules. 

Combined with the variance results in Table 10, the recommended preparation process was A1B2C2D3. Combining the results, the parameters of the two groups of the recommended preparation process were the same. Therefore, the preferred preparation process parameters of the UV primer microcapsules were obtained as follows: the wall-core mass ratio was 1:0.60, the emulsifier HLB value was 10.04, the microcapsule reaction temperature was 70 °C, and the microcapsule reaction time was 3.0 h.

The single-factor test was carried out to finally determine the best process for preparing the UV primer microcapsules, based on the superior process described above, with the wall-core mass ratio, which has a large, combined effect, as the variable. Based on the wall-core mass ratio of 1:0.60 as the preferred parameter, the wall-core mass ratios in the single-factor test were 1:0.50, 1:0.55, 1:0.60, 1:0.65, and 1:0.70.

Table 11 shows the yield and encapsulation rate of the UV primer microcapsules in the single-factor test. The yield and encapsulation rate of the microcapsules in sample 10 reached the highest levels (34.48% and 20.03%, respectively). This is due to the fact that when the wall-core mass ratio was larger, the higher ratio of the wall material could provide better and more comprehensive encapsulation of the core material, resulting in the higher yield and encapsulation rate of the microcapsules. Therefore, microcapsules in sample 10 were the best microcapsules prepared in the single-factor test. The best process for the preparation of UV primer microcapsules was as follows: the wall-core mass ratio was 1:0.50, the emulsifier HLB value was 10.04, the microcapsule reaction temperature was 70 °C, and the microcapsule reaction time was 3.0 h.

#### 3.2.2. Microscopic Morphology of UV Primer Microcapsules

**Figure 2:**
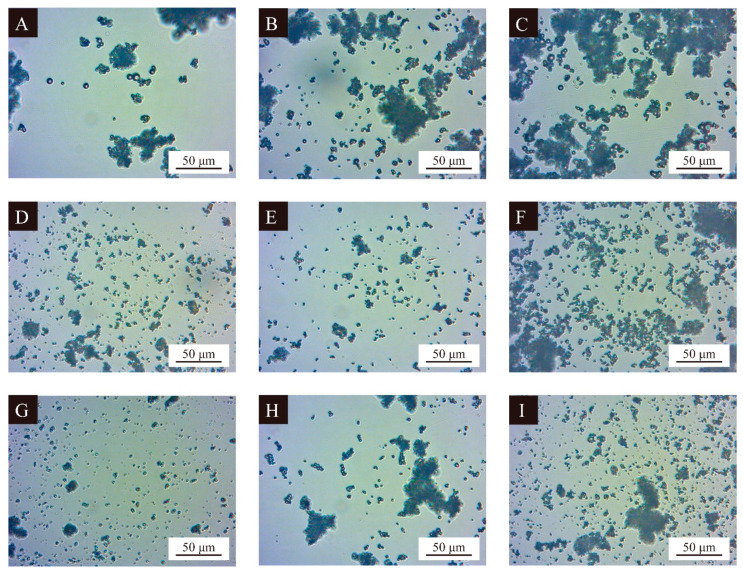
Optical microscopy of UV primer microcapsules in orthogonal test: (**A**–**I**) Sample 1–9.

Figure 2 shows optical morphologies of the microcapsules in the orthogonal tests. The particle sizes of the three groups of samples, 1, 2, and 3, with wall-core mass ratios of 1:0.60, were larger than those of the other six groups, indicating that larger wall-core mass ratios were favorable for the generation of microcapsules with larger particle sizes. This is because under the same mass of wall materials, less of the core material is mixed with wall solution when forming the homogeneous emulsion; as such, the wall materials can better encapsulate the core materials to form the microcapsules with larger particle sizes when synthesized. The sticky condition of samples 3, 6, and 9 was more serious, indicating that when the HLB value of the emulsifier was larger, it was not conducive to the dispersion of the microcapsules when they were formed, which indirectly affected the subsequent distribution of the microcapsules in the coating. 

**Figure 3:**
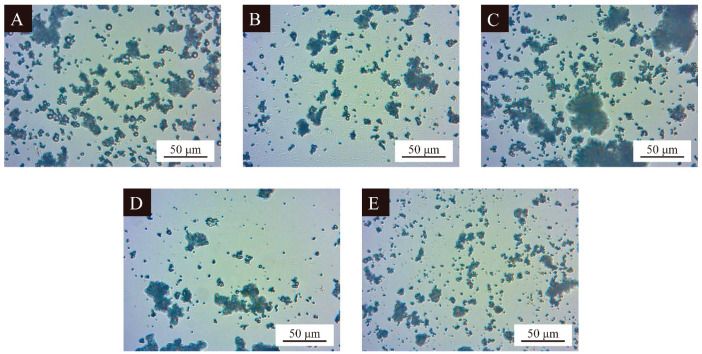
Optical microscopy of UV primer microcapsules’ samples in single-factor test: (**A**–**E**) Sample 10–14.

Figure 3 shows the distribution morphology of the microcapsule powders and reflects the severity of the agglomeration phenomenon of the microcapsule powders, as well as the microcapsules’ encapsulation. The optical microscope figures of the microcapsule samples prepared in the single-factor test, in which the particle sizes of samples 10 and 11 were moderate, the morphology was smooth and rounded, and with the gradual decrease of the wall-core mass ratio, show that the particle size of the microcapsules gradually decreased and the morphology gradually became worse, which indicates that when the wall-core mass ratio was too low, the conditions were not conducive to the preparation of microcapsules in UV primers.

**Figure 4:**
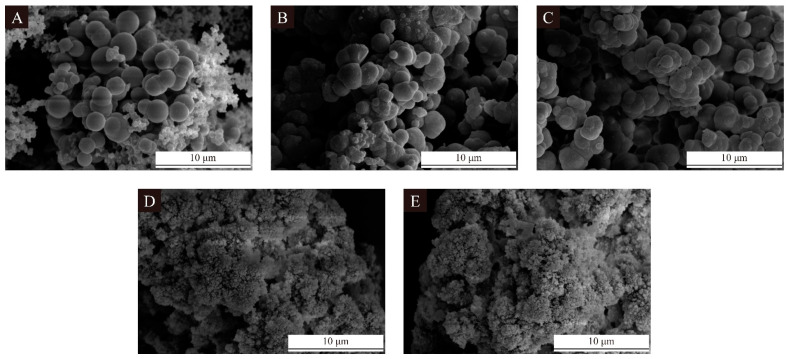
SEM of UV primer microcapsules in single-factor test: (**A**–**E**) Sample 10–14.

Figure 4 shows the SEM images of the microcapsules in the single-factor test. It can be seen from Figure 4A–C that when the wall-core mass ratio was less than 1:0.60, the microcapsules’ spherical structure was obvious and numerous, and the particle size was also relatively large. As the wall-core mass ratio continued to decrease to 1:0.65 and below, as shown in Figure 4D,E, the particle size of the microcapsules decreased dramatically and the sticky condition was very serious. This is because the microcapsule wall material was melamine formaldehyde resin, which has a high viscosity, and the main components of the core material, epoxy acrylic resin and polyester acrylic resin, also have a high viscosity. As the wall-core mass ratio decreased, the relative proportion of the core material in the mixed emulsion increased, resulting in a deterioration of the fluidity of the whole emulsion after mixing of the wall materials and the core materials and impeding an adequate contact between the wall and the core material in the synthesis of the microcapsules. This led to a sharp decrease in the particle size of the synthesized microcapsules and caused a serious sticky condition, meaning that it was difficult for the core material to exist in the form of single particles.

**Figure 5:**
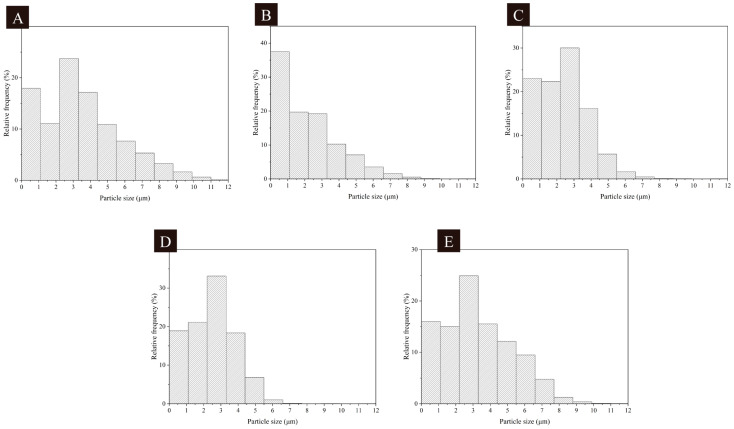
Particle size distribution of single-factor test microcapsules: (**A**–**E**) Sample 10–14.

Figure 5 shows the particle size distribution of the microcapsules’ samples prepared by the single-factor test. The microcapsules in sample 10 had a particle size ranging from 2.0 μm to 4.0 μm, the microcapsules in sample 11 had particle sizes ranging from 1.0 μm to 3.0 μm, the microcapsules in sample 12 had particle sizes ranging from 1.0 μm to 4.0 μm, the microcapsules in sample 13 had particle sizes ranging from 1.0 μm to 4.0 μm, and the microcapsules in sample 14 had particle sizes ranging from 2.0 μm to 4.0 μm. Samples 10 and 13 showed a more uniform distribution of particle size. The particle size distributions of the microcapsules were all uniform, and the microcapsules were all small in size. The particle size distribution was more centralized for the samples with a wall-core mass ratio close to 1:0.60.

#### 3.2.3. Chemical Composition Analysis of UV Primer Microcapsules

**Figure 6:**
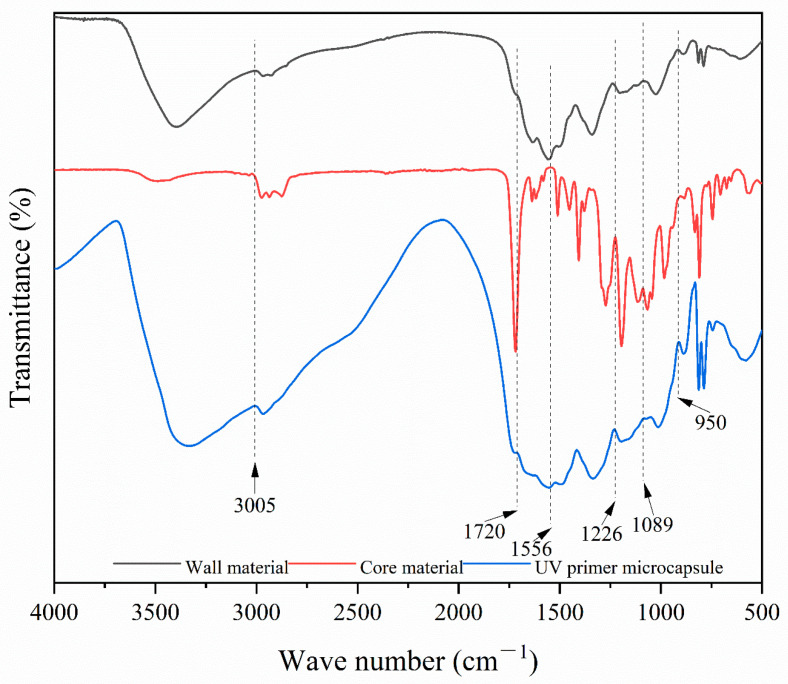
Infrared spectrum of UV primer microcapsules.

Figure 6 shows the infrared spectrum of the UV primer microcapsules. Absorption peaks at 1089 cm^−1^ and 1720 cm^−1^ were C-O-C and C=O stretching vibration peaks, respectively, and the peak at 1556 cm^−1^ was an -NH- bending vibration peaks. These are characteristic peaks belonging to melamine formaldehyde resin, and they appeared in the absorption curves of both the wall material and the microcapsules. The melamine formaldehyde resin of the wall material had been successfully prepared and existed in the microcapsules. An epoxy group stretching vibration peak of epoxy acrylic resin, which was the main component of the core material, was at 950 cm^−1^, and a C-O stretching vibration peak and a C-H stretching vibration peak were at 1226 cm^−1^ and 3005 cm^−1^, respectively. These were the common characteristic peaks of polyester acrylic resin, trihydroxy methacrylate, and TriMet acrylate, respectively, and they appeared in the absorption curves of both the core material and the microcapsule. By analyzing the chemical composition of the microcapsules, the chemical composition of the core material was not destroyed and existed in the microcapsule, which proved that the UV primer microcapsules were successfully prepared. It was found that the wall material successfully polymerized to encapsulate the core material. The microcapsules can provide a better self-healing effect, considering the compatibility between the microcapsules and the UV primer, because of the consistency of the microcapsules core material and the UV primer as well as the ability of the microcapsules wall material, melamine formaldehyde resin, to insulate the exterior and protect the core materials.

Combining the results of the above analysis, it was clear that sample 10 was the best UV primer microcapsules prepared by the single-factor test. As such, sample 10 was added to the UV primer in different quantities to prepare the coating.

### 3.3. UV Primer Coating Property

#### 3.3.1. UV Primer Coating Morphology

**Figure 7:**
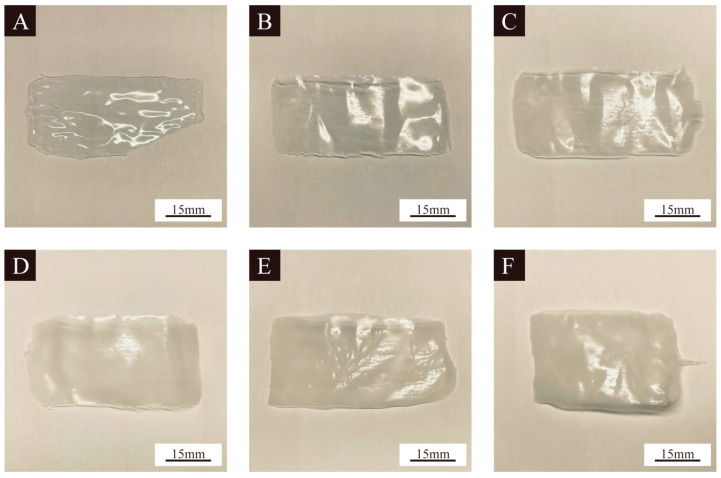
Morphology of UV primer coatings with different additions of UV primer microcapsules: (**A**–**F**) 0%, 2.0%, 4.0%, 6.0%, 8.0%, and 10.0%, respectively.

Figure 7 shows the morphologies of the coating with different additions of the UV primer microcapsules from sample 10. Because the UV primer itself was colorless, the coating without the addition of UV primer microcapsules was clear and transparent. The UV primer microcapsules were a white powder; with the increasing addition of the microcapsules, the coating became more and more white and gradually not transparent. With the 2.0% addition, the transparency of the coating underwent almost no change. When the 4.0% addition, the coating was slightly white and became translucent. When the addition reached 6.0% and above, the coating turned completely white and was no longer transparent. 

**Figure 8:**
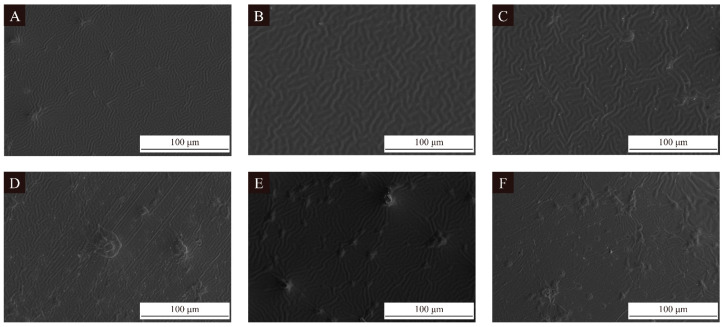
SEM images of UV primer coatings with different additions of UV primer microcapsules: (**A**–**F**) 0%, 2.0%, 4.0%, 6.0%, 8.0%, and 10.0%, respectively.

Figure 8 shows the SEM images of different additions of the sample 10 UV primer microcapsules. When no microcapsules were added, the surface of coating was smooth and delicate. After the addition of UV primer microcapsules, irregular lines appeared on the coating surface. With the 2.0% and 4.0% additions of UV primer microcapsules, the most obvious lines appeared on the surface of the coating. This is because the spherical microcapsules affect the fluidity of the coating and, in the process of rapid curing of the coating by UV irradiation, the coating is unable to be completely leveled, resulting in wrinkles and forming lines. When the addition of UV primer microcapsules reached 6.0% and above, the surface of the coating had crater-like depressions; the number of depressions gradually increased with the increase of the addition of UV primer microcapsules in the coating. It was difficult for the microcapsules to disperse homogeneously, which led to the surface of the coating producing a significant difference in transparency and the formation of irregular bumps and depressions. 

The transparency weakening of the coating decreases to a great extent, which was unrequired in use. The transparency weakening reduces the added value of the surface aesthetics of the coating. Roughness and pits in the coating at the microscopic level not only reduce the surface aesthetics of the coating, but also affect the optical and mechanical properties of the coating, making the coating stress distribution uneven and leading to cracking and blistering of the coating, thus reducing the service life of the coating. The addition of microcapsules in the coating should not be excessive.

#### 3.3.2. Chemical Composition Analysis of UV Primer Coating

**Figure 9:**
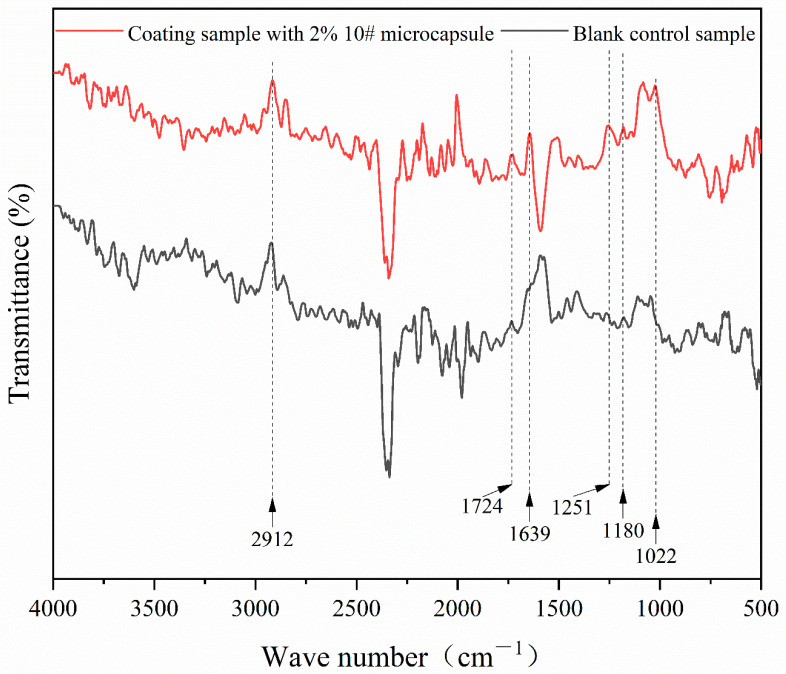
Infrared spectrum of UV primer coating.

Figure 9 shows the infrared spectrum of the coating with the sample 10 microcapsules added and the coating of the blank control group. There were C-O-C, C-N, and C=O telescopic vibration peaks at 1022 cm^−1^, 1251 cm^−1^, and 1639 cm^−1^, respectively, that belonged to the wall material. These peaks were characteristic of melamine formaldehyde resin, and they only appeared in the absorption curves of the coating with added microcapsules, indicating that the wall material only existed in the coating with added microcapsules and the chemical composition remained intact. The C-O and C=O stretching vibration peaks at 1180 cm^−1^ and 1724 cm^−1^, respectively, are common characteristic peaks belonging to polyester acrylic resin, trihydroxy methacrylate, and TriMet acrylate, which were the main components of the core materials and the UV primer. The C-H stretching vibration peaks of epoxy acrylic resin, which were the main component in the core materials and the UV primer, were at 2912 cm^−1^, and they appeared in both absorption curves. The addition of the microcapsules had no effect on the coating-forming reaction process of the curing of UV primer, proving that the microcapsules could be stabilized and that they existed in the UV primer.

#### 3.3.3. Optical Properties of UV Primer Coating

Table 12 shows the chromaticity values and color difference values of the coatings with different additions of the sample 10 UV primer microcapsules. The *L* values of the different coatings were around 80, which indicated that the overall brightness of the coating was relatively bright. At the same time, the *L* values of different coatings showed an overall decreasing trend with the increasing addition of UV primer microcapsules, which indicated that the coatings were gradually becoming darker. The *b* values of the different coatings were positive and gradually increased with the addition of UV primer microcapsules, indicating that the coating color was more yellow. The color difference of the UV primer coating showed a rising trend, reaching a maximum value of 6.12 when the addition of UV primer microcapsules was 10.0%; this is because the excess of microcapsules makes the coating completely white. 

**Figure 10:**
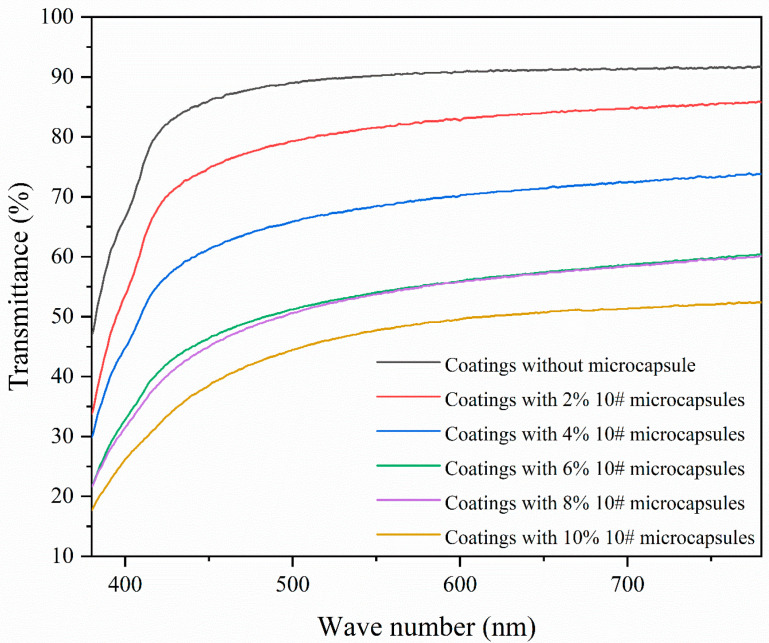
The transmittance of UV primer coatings with different addition of UV primer microcapsules.

Table 13 shows the gloss and transmittance of coatings with different additions of UV primer microcapsules. Figure 10 shows the variation of transmittance of coating with different additions of UV primer microcapsules. The transmittance of different coatings showed an overall decreasing trend with the increase of the addition of UV primer microcapsules, indicating that the light reflection ability of the coating gradually weakened. This is because the addition of UV primer microcapsules makes the coating uneven, which leads to a decrease in the transmittance. The transmittance of the coating was inversely proportional to the addition of the UV primer microcapsules. The transmittance of the coating without the addition of UV primer microcapsules was 87.51%, and when the addition of UV primer microcapsules was 4.0% and below, the transmittance of the coating was slightly decreased. When the addition of UV primer microcapsules reached 6.0% and above, the transmittance of the coating decreased abruptly, down to 45.37%, which was consistent with the morphological changes of the coating, and the white microcapsules caused obstruction to the transmittance.

#### 3.3.4. Mechanical Properties of UV Primer Coating

The effect of the addition of UV primer microcapsules at different additions of the sample 10 microcapsules on the elongation at break of the coating is shown in Table 14. The elongation at break of the UV primer coating showed an increasing then decreasing trend with the increase of microcapsule addition. When adding sample 10 microcapsules at a 2.0% addition, the elongation at break of UV primer coating was up to 3.62% and then began to decline. The values of elongation at break of different coatings were not large, which was directly related to the high brittleness of UV coatings after curing. Therefore, the addition of UV primer microcapsules should not be too high, otherwise it will cause a large negative impact on the mechanical properties of the coating.

Table 15 shows the roughness of coating with different addition of sample 10 UV primer microcapsules. When no microcapsules were added, the roughness of the coating was extremely low, only 0.030 μm; with the addition of microcapsules, the roughness increased sharply, up to 0.442 μm.

#### 3.3.5. Self-Healing Property of UV Primer Coating

**Figure 11:**
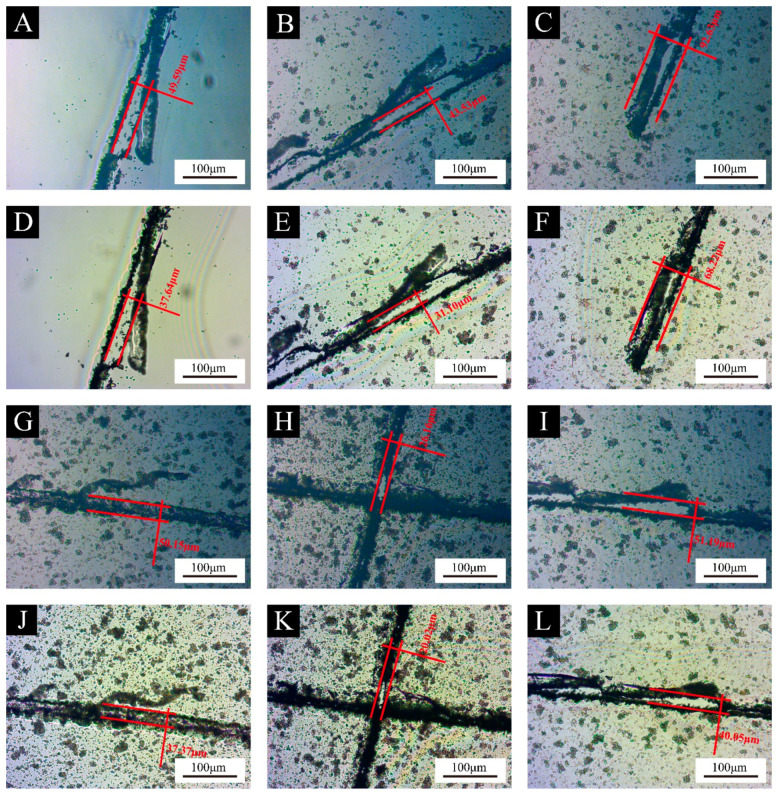
Comparison of cracks before and after self-healing of UV primer coatings with different additions of sample 10 UV primer microcapsules. Before self-healing: (**A**) without microcapsules, (**B**) 2.0%, (**C**) 4.0%, (**G**) 6.0%, (**H**) 8.0%, (**I**) 10.0%; after self-healing: (**D**) without microcapsules, (**E**) 2.0%, (**F**) 4.0%, (**J**) 6.0%, (**K**) 8.0%, and (**L**) 10.0%, respectively.

The width of the cracks of the coating with different additions of UV primer microcapsules was reduced, which proved that the UV primer microcapsules positively affected the self-healing property of the UV primer coating. The self-healing property of the coatings with different additions of UV primer microcapsules is shown in Figure 11.

When external factors caused the microcapsules to break, the UV primer within the microcapsules was released, filling the microcracks in the surrounding area. When exposed to natural light, the outflow core materials gradually cure, which not only heals the microcracks but also allows the coating to somewhat return to its previous level of properties before cutting. It can be seen from Figure 8 that the dispersion of microcapsules was getting worse as the addition of microcapsules increased. In the future, the dispersion of microcapsules in the coating can be improved by using suitable dispersants. By improving the preparation technology, such as improved formulation of the microcapsule wall materials, the core microcapsules can be better dispersed in the coating to uniformly act as a self-healing agent and the property of the core materials to act can be better maintained before the rupture of the microcapsules and the UV primer. The self-healing rate of the coating with different additions of UV primer microcapsules is shown in Table 16. The self-healing rate of the coating with the addition of sample 10 microcapsules reached a maximum of 28.56% at the 2.0% addition, which was 4.46% higher than that of the blank UV primer, an improvement rate of 18.51%. Based on our previous research, the self-healing rate of the best sample for coatings with 4.0% of the best addition of microcapsules was 24.53%. The self-healing rate of UV topcoat microcapsules increased by 5.34% compared to that without microcapsules [36]. Compared to the previous research [36], the self-healing rate with the 2.0% addition of UV primer microcapsules was higher. This indicates that for the UV primer microcapsules prepared in this test to achieve the self-healing property of the primer, a low addition of microcapsules is more significant compared to the topcoat microcapsules. Subsequently, the self-healing rate began to decline, and decreased to 23.48% and 21.76% with the addition of UV primer microcapsules at 8.0% and 10.0%, respectively; these were lower than the self-healing rate of the coating without the UV primer microcapsules. This is because the coating itself has a certain degree of toughness; after being damaged over time, the microcracks can gradually recover. The core material of the prepared sample 10 microcapsules was the same as the coating, so when the appropriate addition of microcapsules is added to the coating, it can enable a certain degree of supplementation and healing, and thus the self-healing rate increased. When the addition of UV primer microcapsules was too large, the flow of the core material was obstructed and the brittleness of the coating was increased, resulting in a decrease in the self-healing rate, which was even lower than that of the coating without the addition of microcapsules. Therefore, a directional release approach should be considered in future self-healing microcapsule research to endow the microcapsule cores with the property to perform directional flow toward the microcracks. By reducing the obstruction of the flow of the core materials by the high-density microcapsules, the microcapsules can be made more efficient in healing the coatings.

## 4. Conclusions

UV primer microcapsules with melamine formaldehyde resin as the wall material and the UV primer as the core material were prepared and optimized using orthogonal and single-factor tests. The effects of wall-core mass ratio, emulsifier HLB value, microcapsule reaction temperature, and microcapsule reaction time on microcapsules yield and encapsulation rate were investigated. The factor most affecting the microcapsules yield and encapsulation rate was the wall-core mass ratio, and the preferred preparation process of the UV primer microcapsules was as follows: a wall-core mass ratio of 1:0.50, an emulsifier HLB value of 10.04, a microcapsule reaction temperature of 70 °C, and a microcapsule reaction time of 3.0 h. The results showed that the comprehensive properties of the coating with the addition of sample 10 UV primer microcapsules were better than that of the blank control group, and the addition of microcapsules increased the color difference, lowered the gloss, and weakened the transmittance. The elongation at break of the coating showed a trend of first increasing and then decreasing, the roughness gradually increased, and the self-healing rate showed a trend of first increasing and then decreasing. When the addition of sample 10 microcapsules was 2.0%, the coating had better comprehensive properties, with a color difference Δ*E* of 1.71, gloss of 106.63 GU, transmittance of 78.80%, elongation at break of 3.62%, roughness of 0.204 μm, and a self-healing rate of 28.56%, which was 4.46% higher than that of the blank control group, with an enhancement rate of 18.51%. There is a need for further research on the improvement of the self-healing property of UV primers. Taking the self-healing microcapsules with the UV primer itself as the core material further broadens the choice of core material and provides an idea of preparing self-healing microcapsules relative to previous studies.

## Figures and Tables

**Table 1:** Test materials.

Experimental Materials	Molecular Mass (g/mol)	CAS	Producer
37% formaldehyde	30.03	50-00-0	Wuxi Yatai United Chemical Co., Ltd., Wuxi, China
Melamine	126.12	108-78-1	Nanjing Quanlong Biotechnology Co., Ltd., Nanjing, China
Triethanolamine	149.19	102-71-6	Nanjing Houxin Biotechnology Co., Ltd., Nanjing, China
Span-20	346.459	133-39-2	Nanjing Houxin Biotechnology Co., Ltd., Nanjing, China
Triton X-100	646.85	9002-93-1	Hangzhou Chicheng Pharmaceutical Technology Co., Ltd., Hangzhou, China
Absolute ethanol	46.07	64-17-5	Sichuan Kelun Pharmaceutical Co., Ltd., Chengdu, China
UV primer	-	-	Guangdong Huankai Microbial Technology Co., Ltd., Guangzhou, China
Citric acid monohydrate	210.139	5949-29-1	Jinan Xiaoshi Chemical Co., Ltd., Jinan, China

**Table 2:** Test equipment.

Equipment	Model	Manufacturer
Water bath	LC-OB-5L	Shangpin Bense Smart Home Co., Ltd., Zaozhuang, China
Scanning electron microscope	Zeiss Sigma 300	FEI Company, Hillsboro, OR, USA
Powder tablet press	HY-12	Tianjin Tianguang Optical Instrument Co., Ltd., Tianjin, China
Infrared spectrometer	VERTEX 80V	Germany BRUKER Co., Ltd., Karlsruhe, Germany
Color difference meter	3nhYS3010	Shenzhen Linshang Technology Co., Ltd., Shenzhen, China
Glossmeter	3nhYG60S	Shenzhen Linshang Technology Co., Ltd., Shenzhen, China
Ultraviolet spectrophotometer	U-3900	Hitachi High-Tech Co., Ltd., Beijing, China
Universal mechanical testing machine	5000N	Zhejiang Wanxiong Instrument Manufacturing Co., Ltd., Ningbo, China
Coating roughness tester	SJ-411	Shanghai Taiming Optical Instrument Co., Ltd., Shanghai, China
Single-lamp curing machine	620#	Huzhou Tongxu Machinery Equipment Co., Ltd., Huzhou, China

**Table 3:** Orthogonal test factors and levels.

Levels	Factor A Wall-Core Mass Ratio	Factor B Emulsifier HLB Value	Factor C Temperature (°C)	Factor D Time (h)
1	1:0.60	8.60	60	1.0
2	1:0.70	10.04	70	2.0
3	1:0.80	13.40	80	3.0

**Table 4:** Orthogonal test schedule.

Sample (#)	Factor A Wall-Core Mass Ratio	Factor B Emulsifier HLB Value	Factor C Temperature (°C)	Factor D Time (h)
1	1:0.60	8.60	60	1.0
2	1:0.60	10.04	70	2.0
3	1:0.60	13.40	80	3.0
4	1:0.70	8.60	70	3.0
5	1:0.70	10.04	80	1.0
6	1:0.70	13.40	60	2.0
7	1:0.80	8.60	80	2.0
8	1:0.80	10.04	60	3.0
9	1:0.80	13.40	70	1.0

**Table 5:** Materials for orthogonal test.

Sample (#)	Triton X-100 (g)	Span-20 (g)	Ethanol (mL)	Primer (g)	Formaldehyde (g)	Melamine (g)	Deionized Water (mL)
1	0.00	0.30	78.90	4.40	9.01	4.00	20.00
2	0.08	0.22	78.90	4.40	9.01	4.00	20.00
3	0.30	0.00	78.90	4.40	9.01	4.00	20.00
4	0.00	0.30	78.90	4.40	7.72	3.43	17.50
5	0.08	0.22	78.90	4.40	7.72	3.43	17.50
6	0.30	0.00	78.90	4.40	7.72	3.43	17.50
7	0.00	0.30	78.90	4.40	6.76	3.00	15.00
8	0.08	0.22	78.90	4.40	6.76	3.00	15.00
9	0.30	0.00	78.90	4.40	6.76	3.00	15.00

**Table 6:** Materials for single-factor test.

Sample (#)	Triton X-100 (g)	Span-20 (g)	Ethanol (mL)	Primer (g)	Formaldehyde (g)	Melamine (g)	Deionized Water (mL)
10	0.08	0.22	78.90	4.40	10.81	4.80	24.00
11	0.08	0.22	78.90	4.40	9.83	4.36	21.82
12	0.08	0.22	78.90	4.40	9.01	4.00	20.00
13	0.08	0.22	78.90	4.40	8.32	3.69	18.46
14	0.08	0.22	78.90	4.40	7.72	3.43	17.50

**Table 7:** Analysis on the yield rate of microcapsules by orthogonal test.

Sample (#)	Factor A Wall-Core Mass Ratio	Factor B Emulsifier HLB Value	Factor CTemperature (°C)	Factor DTime (h)	*P* (%)
1	1:0.60	8.60	60	1.0	22.17
2	1:0.60	10.04	70	2.0	26.42
3	1:0.60	13.40	80	3.0	32.22
4	1:0.70	8.60	70	3.0	27.65
5	1:0.70	10.04	80	1.0	22.32
6	1:0.70	13.40	60	2.0	21.29
7	1:0.80	8.60	80	2.0	11.86
8	1:0.80	10.04	60	3.0	23.38
9	1:0.80	13.40	70	1.0	15.32
Mean value 1	26.937	20.560	22.280	19.937	
Mean value 2	23.753	24.040	23.130	19.857	
Mean value 3	16.853	22.943	22.133	27.750	
Range	10.084	3.480	0.997	7.893	
Order of influencing factors	A > D > B > C				
Optimal level	A1	B2	C2	D3	
Recommended preparation process	A1B2C2D3				

**Table 8:** Variance analysis table of yield rate.

Factors	Quadratic Sum	Free Degree	F-Ratio	F-Critical Value	Significance
A	159.417	2	2.101	4.460	
B	18.993	2	0.250	4.460	
C	1.737	2	0.023	4.460	
D	123.359	2	1.626	4.460	
Error	303.51	8			

**Table 9:** Analysis of the results of the orthogonal test on microcapsules encapsulation rate.

Sample (#)	Factor A Wall-Core Mass Ratio	Factor B Emulsifier HLB Value	Factor CTemperature (°C)	Factor DTime (h)	*P_e_* (%)
1	1:0.60	8.60	60	1.0	48.15
2	1:0.60	10.04	70	2.0	50.85
3	1:0.60	13.40	80	3.0	48.01
4	1:0.70	8.60	70	3.0	46.06
5	1:0.70	10.04	80	1.0	45.14
6	1:0.70	13.40	60	2.0	42.35
7	1:0.80	8.60	80	2.0	45.83
8	1:0.80	10.04	60	3.0	47.67
9	1:0.80	13.40	70	1.0	43.11
Mean value 1	49.003	46.680	46.057	45.467	
Mean value 2	44.517	47.887	46.673	46.343	
Mean value 3	45.537	44.490	46.327	47.247	
Range	4.486	3.397	0.616	1.780	
Order of influencing factors	A > B > D > C				
Optimal level	A1	B2	C2	D3	
Recommended preparation process	A1B2C2D3				

**Table 10:** Variance analysis table of encapsulation rate.

Factors	Quadratic Sum	Free Degree	F-Ratio	F-Critical Value	Significance
A	33.188	2	2.358	4.460	
B	17.789	2	1.264	4.460	
C	0.573	2	0.041	4.460	
D	4.753	2	0.338	4.460	
Error	56.30	8			

**Table 11:** Microcapsules yield rate and encapsulation rate of single factor test.

Sample (#)	Factor A Wall-Core Mass Ratio	*P* (%)	*P_e_* (%)
10	1:0.50	34.48	20.03
11	1:0.55	32.33	19.86
12	1:0.60	30.67	19.55
13	1:0.65	29.98	18.78
14	1:0.70	27.85	18.57

**Table 12:** Effect of different addition of sample 10 microcapsules on the chromaticity and color difference values of coatings.

Sample 10 UV Primer Microcapsules (%)	*L*	*a*	*b*	Δ*E*
0	82.13	0.40	0.03	-
2.0	80.53	0.67	0.57	1.71
4.0	80.73	0.70	1.03	1.75
6.0	80.07	0.50	1.17	2.36
8.0	79.03	0.67	1.47	3.43
10.0	76.97	0.90	3.27	6.12

**Table 13:** Effect of different addition of sample 10 microcapsules on the gloss and transmittance of coatings.

Sample 10 UV Primer Microcapsules (%)	Gloss (GU)			Transmittance (%)
	20°	60°	85°	
0	162.47	165.93	110.33	87.51
2.0	91.63	106.63	82.77	78.80
4.0	71.73	95.83	73.33	66.39
6.0	48.87	102.77	87.60	52.39
8.0	36.07	80.00	74.33	51.84
10.0	43.57	82.83	72.60	45.37

**Table 14:** Effect of different additions of sample 10 microcapsules on the elongation at break of coatings.

Sample 10 UV Primer Microcapsules (%)	Elongation at Break (%)
0	2.67
2.0	3.62
4.0	1.19
6.0	1.53
8.0	1.36
10.0	0.99

**Table 15:** Effect of different additions of sample 10 microcapsules on the roughness of coatings.

Sample 10 UV Primer Microcapsules (%)	Roughness (μm)
0	0.030
2.0	0.204
4.0	0.232
6.0	0.234
8.0	0.287
10.0	0.442

**Table 16:** The self-healing rate of coatings with different additions of sample 10 microcapsules.

Sample 10 UV Primer Microcapsules (%)	Crack Width (μm)		Self-Healing Rate (%)
	After Cutting	One Week Later	
0	49.59	37.64	24.10
2.0	43.53	31.10	28.56
4.0	92.63	68.22	26.35
6.0	50.15	37.37	25.49
8.0	26.16	20.02	23.48
10.0	51.19	40.05	21.76

## Data Availability

Data are contained within the article.
